# Frozen section accurately allows pathological characterization of endometrial cancer in patients with a preoperative ambiguous or inconclusive diagnoses: our experience

**DOI:** 10.1186/s12885-019-6318-5

**Published:** 2019-11-12

**Authors:** A. Santoro, A. Piermattei, F. Inzani, G. Angelico, M. Valente, D. Arciuolo, S. Spadola, M. Martini, F. Fanfani, A. Fagotti, V. Gallotta, G. Scambia, G. F. Zannoni

**Affiliations:** 1 Unità di Gineco-Patologia e Patologia Mammaria, Dipartimento Scienze della Salute della Donna, del Bambino e di Sanità Pubblica, Fondazione Policlinico Universitario A. Gemelli IRCCS, Roma, Italy; 2 Unità di Ginecologia Oncologica, Dipartimento Scienze della Salute della Donna, del Bambino e di Sanità Pubblica, Fondazione Policlinico Universitario A. Gemelli IRCCS, Roma, Italy; 3 Istituto di Clinica Ostetrica e Ginecologica, Università Cattolica del Sacro Cuore, Roma, Italy; 4 Istituto di Anatomia Patologica, Università Cattolica del Sacro Cuore, Roma, Italy; 5 UOC di Anatomia Patologica, Dipartimento Scienze della Salute della Donna, del Bambino e di Sanità Pubblica, Fondazione Policlinico Universitario A. Gemelli IRCCS, Roma, Italy

**Keywords:** Endometrial carcinoma, Frozen section, Intraoperative surgical staging

## Abstract

**Background:**

The aim of this study was to assess the agreement rate between intraoperative evaluation (IOE) and final diagnosis (FD) in a series of surgically resected endometrial carcinoma (EC), with a preoperative ambiguous or inconclusive diagnosis by endometrial biopsies and imaging.

**Methods:**

A retrospective study was performed selecting patients who underwent surgery with IOE for suspected EC at our institution from 2012 to 2018. A K coefficient was determined with respect to the histotype, tumor grade, myometrial infiltration and cervical involvement.

**Results:**

Data analysis has been performed on 202 women. The IOE evaluation was distributed as Endometrioid (*n* = 180) and Non-Endometrioid (*n* = 22). The comparison between the frozen section (FS) and the definitive histological subtype showed an overall agreement rate of 93,07% (k = 0.612) and an agreement of 97.2% for Endometrioid vs 59% for Non-Endometrioid tumors. The FIGO system grading was the same in 91,1% of patients, none was upgraded and in 8,9% downgraded. Observed agreements were 89,11% and 95,54% for myometrial and cervical involvement, respectively.

**Conclusions:**

The good agreement between intraoperative grading, myometrial invasion and their histological definition on permanent sections highlights that FS is a good predictor for surgical outcome, in particular in presence of a preoperative ambiguous or inconclusive diagnostic evaluation.

## Background

Endometrial carcinoma (EC) is the most common gynecological malignant neoplasia in industrialized countries and its incidence has been constantly increasing [1]. Approximately 85% of cases are diagnosed at an early-stage (International Federation of Gynecology and Obstetrics: FIGO I and II) while 15% are diagnosed in advanced stage (FIGO III and IV) [2]. It is well documented that surgical staging and treatment represent the first approach for the affected patients.

Information regarding tumoral grading and histotype can be obtained in most cases from preoperative diagnostic endometrial biopsies or curettage however, intraoperative pathological examination (IOE) increases the sensitivity and specificity for the patient risk classification and, thus, plays a fundamental role in the evaluation of surgical decision [3–5].

In fact, before being compared with the final result, the frozen endometrial tissue obtained during surgery provides an important prognostic tool for the prediction of the final diagnosis as well as for the decision of final extended surgical staging, thus identifying high risk-patients requiring pelvic/para-aortic lymphadenectomy.

Moreover, according to literature data, the IOE should always be used in cases where the preoperative diagnosis is not conclusive. However there are many controversies about the use of IOE in the characterization of EC, with conflicting results regarding the intraoperative accuracy in the evaluation of grading and myometrial invasion, IFS accurately identified 90% of the patients requiring pelvic/para-aortic lymphadenectomy.

IOE is also important for the evaluation of lymh-nodes status; in fact the use of sentinel node, as intraoperative surgical staging tool, has been implemented in the last years in order to avoid staging lymphadenectomy in low-risk EC patients according to ‘Mayo criteria’ grade 1 or 2 disease, < 50% myometrial invasion, and tumor diameter < 2 cm) [6]. On the other hand, in high risk patients (endometrioid grade 3, clear cell, serous, and carcinosarcoma) the same procedure has no impact in the choice of adjuvant therapy and more studies are still needed to determine if SLN mapping could replace total lymphadenectomy [7].

Hence, there is a need to evaluate the accuracy of the intraoperative endometrial sampling in order to early define the histopathological prognostic factors and deploy a strategy to reduce and eliminate discrepancies between frozen section examination and final report. The purpose of this study was to evaluate if the intraoperative histopathological reporting of endometrial cancer could be considered a good prognostic predictor of final histological diagnosis.

## Methods

### Ethics statement

The retrospective study was performed on clinical and pathological data from 202 women with a preoperatively ambiguous vs inconclusive vs suspected histological and instrumental diagnosis for endometrial carcinoma who underwent to surgical staging from January 2012 to December 2018 at the Department of Gynecology, Fondazione ‘Policlinico Gemelli’, Rome, Italy.

The study was approved by the University Ethical Committee for Research and Review Board of the Fondazione ‘Policlinico Gemelli’ and written consent was requested and obtained from all patients before hospitalization.

### Patients selection

In order to evaluate the agreement between intraoperative endometrial sampling and surgical specimen findings, the complete clinical and pathological data from the 202 consecutive patients with a preoperatively ambiguous vs inconclusive vs suspected histological and instrumental diagnosis for endometrial carcinoma and treated at our institution (Department of Gynecology, Fondazione ‘Policlinico Gemelli’, Rome, Italy) were collected.

We considered as hystological ambiguous and/or suspicious lesion:

- a histological preoperative biopsy with a report of endometrial atypical hyperplasia with some features suggestive or suspicious for carcinoma
- a carcinoma being difficult to subtype as low grade versus high grade
- biopsies characterized by extensive necrosis with few scattered frankly malignant cells

In detail, we consider as radiological (MRI) ambiguous lesions cases showing:

- thinning of the myometrium, tumor extension into the cornua, myometrial compression from a polypoid tumor, and presence of leiomyomas or adenomyosis which limited the real entity of tumoral infiltration

### Intraoperative examination (IOE)

Our laboratory uses IOE to obtain informations about the tumor histotype, grade, myometrial invasion (MI) and cervical involvement (CI), especially in cases in which these parameters are preoperatively ambiguous or inconclusive by endometrial biopsies and imaging. IOE is also used to identify those patients with (apparent) low-stage and low-grade endometrioid adenocarcinomas who have adverse prognostic features identified only at operation time.

In our Institution the surgical management changes on the basis of the following IOE pathological parameters:

- high grade histologies
- myoinvasion > 50%
- cervical stromal invasion
- tumor extension > 4 cm

These characteristics, when intraoperatively observed, required pelvic lymphadenectomy.

For all the patients, the uterus, with fallopian tubes and ovaries was removed and submitted as fresh intact surgical sample. Macroscopic examination of the surgical samples was always performed by a pathologist with a high level of expertise in the field of gynecological pathology. In detail, the uterus, was measured in three dimensions and then cut with scissors through its lateral walls from the cervix to the uterine cornua. A mark was made on its anterior half and parallel transverse sections through each half, beginning at the upper level of the endocervical canal to the fundus were performed at 3 to 5 mm intervals to look for possible myometrial invasion foci. The estimated depth of myometrial invasion was reported as lesser or greater than 50% and, after that, a full-thickness incision was made through the tumor and submitted as FS for the intraoperative diagnosis.

Several sections were made also along the endocervical canal in order to evaluate a possible neoplastic cervical involvement.

These operative findings, completed by a grading intraoperative assessment were compared with the final histological report.

### Final pathological report

Surgical samples were fixed with formaldehyde several hours or overnight; then permanent sections (PS) for final histology were performed as follows:

- one section from the anterior half and one from the posterior half of the cervix
- if obvious tumor was present: 3 sections for the neoplasia with the complete uterine wall, one of which including the area of deepest invasion; 1–2 sections from non-neoplastic endometrium
- if no obvious tumor was present: endometrium was sampled entirely
- sections from left and right parametria
- sections from tubes and ovaries
- sections from other pelvic nodes (when pelvic lymphadenectomy has been performed), if they are present

Depending on the histo-morphological characteristics all tumors were grouped in respectively *Endometrioid adenocarcinoma (EA)* and *Non-Endometrioid Carcinoma (NEC)*. In accordance with FIGO recommendations, endometrioid cancers were distinguished in three grades of tumor differentiation: well differentiated (G1), moderately differentiated (G2) and poorly differentiated (G3) [8]. NECs (clear-cells and serous endometrial cancers) were classified as high grade.

Myometrial invasion was reconsidered and definitively estimated on the basis of the extension of neoplastic cells on the entire myometrial thickness and synthetically noted as less or greater than 50%; regarding cervical involvement we evaluated the presence or absence of stromal neoplastic infiltration.

### Statistical analysis

Intraoperative samples and PS were evaluated by pathologists with a specific training in the gynecological field. Descriptive data were expressed as absolute values, relative percentages and mean or median with standard deviations. The agreement rate between the frozen section (FS) and PS was performed using Cohen’s Kappa test (k).

Accurate FS pathology was defined as complete concordance between FS reporting and definitive reporting of definitive sections with regard to histotype, histopathological grade, depth of myometrial invasion (no MI, MI < 50% or MI ≥50%) and CI. Any degree of discordance between the FS and final histopathology was defined as inaccurate FS pathology.

The interpretation of the agreement by Kappa value was performed with the intervals: k < 0, less than chance agreement; k = 0.01–0.20, slight agreement; k = 0.21–0.40, fair agreement; k = 0.41–0.60, moderate agreement; k = 0.61–0.80, substantial agreement; and k = 0.81–0.99, almost perfect agreement. *P*-values less than 0.05 were considered significant. Descriptive and inferential statistics data were analyzed using the R statistical software package (version 3.3.2 for Windows).

## Results

### Histotype

The distribution of intraoperative and definitive tumor histotype is shown in Table 1.

**Table 1:** Intraoperative and Postoperative Surgical Specimen Histotype Comparison

	Final Diagnosis			
Intraoperative Examination		EA	NEC	TOTAL
	EA	175	5	180
	NEC	9	13	22
	TOTAL	184	18	202

Abbreviations: Endometrioid Carcinoma (EA), Non-Endometrioid Carcinoma (NEC)

Our data indicated that from 180 lesions intraoperatively classified as Endometrioid Cancer, 175 were confirmed in final surgical reports as EA; the remaining 5 cases were NECs that were intraoperatively misdiagnosed as EA G3, but this type of misdiagnoses did not impact the classification as ‘high-risk’ cancer.

On the other hand, of the 22 FSs defined as NEC lesions, including serous cancer and clear cell carcinoma, 13 were confirmed in the same class. The remaining misclassified 9 cases were diagnosed on permanent section as EA G3, but this type of misdiagnoses did not impact the classification as ‘high-risk’ cancer.

The statistical analysis of specific Cohen’s kappa values (k = 0.612, substantial agreement, *p* < 0,001, 95% CI, from 0,427 to 0,797) for each histotype was good and we observed a satisfactory overall agreement (93,07%) that was better for Endometrioid than Non-Endometrioid samples (97,2% vs 59%).

**Fig. 1:**
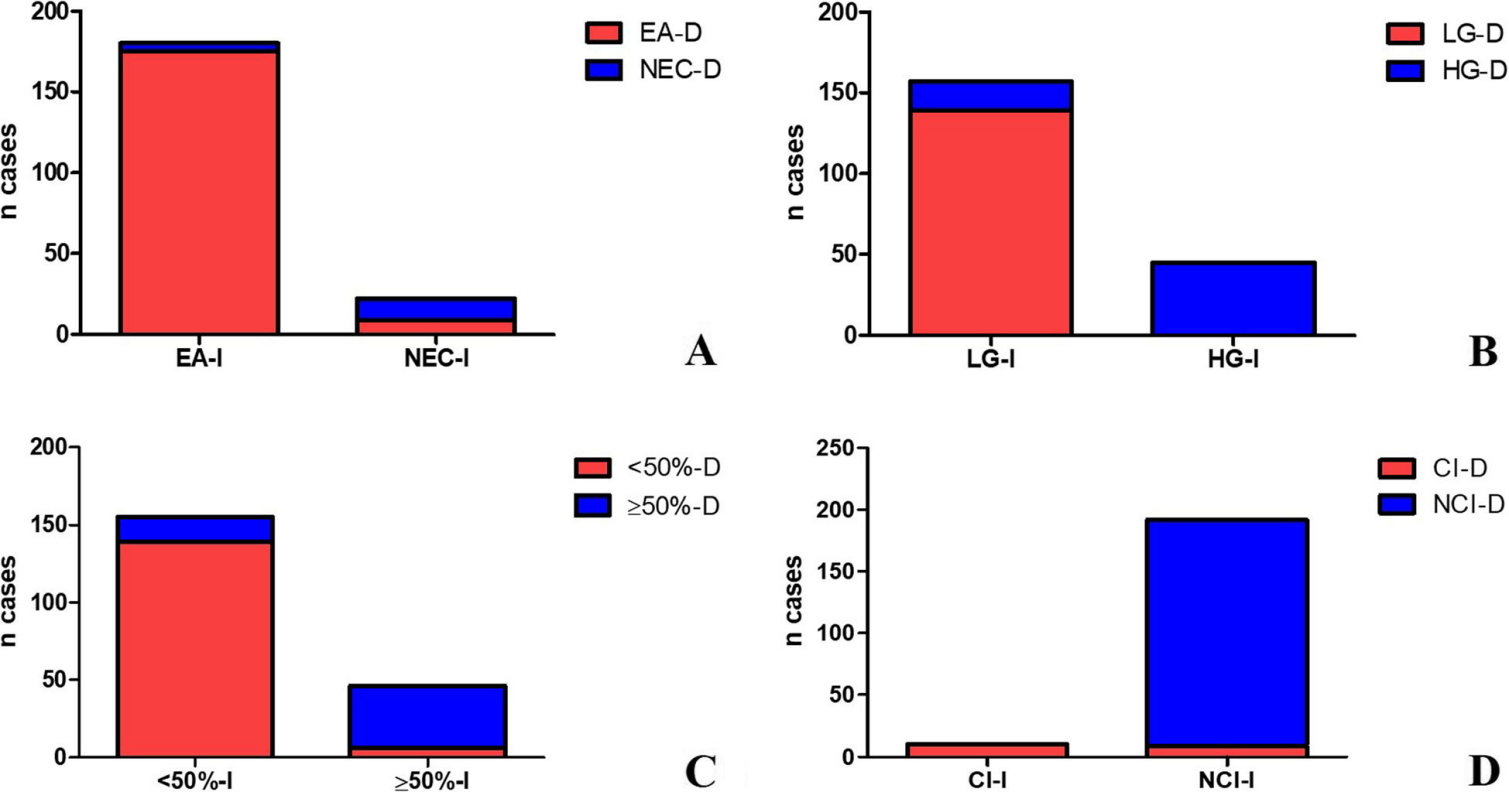
Graphic representation of the differences observed concerning histotype, grading, cervical involvent and myometrial invasion between intraoperative evaluation and final diagnosis. **a**) Histotype: 175/180 lesions considered intraoperatively as Endometrioid Adenocarcinoma (EA) were confirmed in final surgical reports; the remaining 5 cases, with an intraoperative diagnosis of EA, were then diagnosed as Non Endometrioid Adenocarcinoma (NEC) on permanent section; on frozen section, 22 diagnoses of NEC were performed, with 13/22 cases confirmed in definitive evaluation. (*EA-I: Endometrioid Adenocarcinoma-Intraoperative; EA-D: Endometrioid Adenocarcinoma-Definitive; NEC-I: Non Endometrioid Adenocarcinoma-Intraoperative; NEC-D: Non- Endometrioid Adenocarcinoma-Definitive).* **b***) Grade:* 139/157 lesions considered intraoperatively as Low grade were confirmed in final surgical reports; the remaining 18 cases, with an intraoperative diagnosis of Low-grade adenocarcinoma, were then diagnosed as High Grade on permanent section; on frozen section 45 diagnoses of High Grade carcinoma were performed, all confirmed in definitive diagnosis. (*LG-I: Low Grade-Intraoperative; LG-D: Low Grade-Definitive; HG-I: High Grade-Intraoperative; HG-D: High Grade-Definitive*). **c***) Miometrial invasion:* 139/155 lesions evaluated as mioinvasive ≥50% were confirmed in the final diagnosis; the remaining 16 cases, with an intraoperative diagnosis of mioinvasion < 50%, were then diagnosed as mioinfiltrative > 50% on permanent section; on frozen section 47 diagnoses of mioinvasion ≥50% were performed, 41 of which confirmed in definitive diagnosis. *(≥50-I: Mioinvasion ≥ 50% Intraoperative; ≥50-D: Mioinvasion ≥ 50% Definitive; < 50-D: Mioinvasion < 50% Intraoperative; < 50-D: Mioinvasion < 50% Definitive*). **d***) Cervical involvement:* 10 carcinomas intraoperatively considered with cervical invasion were all confirmed in definitive diagnosis; on frozen section, 192 diagnoses of negative cervical involvement were made, 183 of which confirmed in definitive diagnosis. *(CI-I: Cervical Involvement intraoperative; CI-D: Cervical Involvement Definitive; NCI-I: Non Cervical Involvement Intraoperative; NCI-D: Non Cervical Involvement Definitive*)

To further evaluate the histotype discrepancies IOE and final pathology, we calculated the percentage of FSs that were not confirmed in the final diagnosis (2,8% for EA and 41% for NEC). The histotype and grading differences between IOE and final diagnosis are shown in Fig. 1 (a, b).

### Grade

We questioned if intraoperative pathological evaluation was a good indicator for definitive FIGO grade classification. As shown in Table 2, 184/202 patients had not been re-classified and their pathological grade was confirmed as the same. Moreover, while no diagnoses were downgraded, 18 patients (8,9%) were upgraded on the final pathology report.

**Table 2:** Tumor grade on Frozen Sections compared to Permanent Sections

	Final Diagnosis			
Intraoperative Examination		LG	HG	TOTAL
	LG	139	18	157
	HG	0	45	45
	TOTAL	139	63	202

Abbreviations: Low grade (LG), High-grade (HG)

We also calculated the overall agreement for tumor grade that was 91,09% and determined the global k-index (k = 0.775, substantial agreement, *p* < 0,001, 95% CI, from 0.678 to 0.872) that was classified as a good agreement. Figure 1 (b) shows intra and post-operative grade frequencies. Interestingly, when the k index was calculated for each grade, a moderate agreement for G1 (k = 0.4, fair agreement, *p* = 0,099, 95% CI, from 0,210 to 0,598) and G2 grade (k = 0.5, moderate agreement, p = 0,073, 95% CI, from 0,354 to 0,638) and a substantial agreement for G3 (k = 0,76, substantial agreement, p = 0,060, 95% CI, from 0,641 to 0,876) was obtained.

### Myometrial invasion and cervical stromal involvement

The estimation of myometrial invasion (MI) and cervical involvement (CI) was conducted in both FS and final diagnosis in order to find discrepancies.

Starting from 202 cases, the MI was confirmed for 180 patients, while 16 cases were upgraded and six were downgraded in the final report (Table 3). The intra and postoperative MI frequencies are shown in Fig. 1 (c).

**Table 3:** Comparison between Intraoperative and Final Report of MI

	Final Diagnosis			
Intraoperative Examination		MI < 50%	MI ≥ 50%	TOTAL
	MI < 50%	139	16	155
	MI > 50%	6	41	47
	TOTAL	145	57	202

Abbreviations: Miometrial Invasion (MI)

On the basis of the number of observed agreements (89.11% of the observations) and the number of agreements expected by chance (61,65% of the observations), k Cohen’s analysis was performed (k = 0,716, substantial agreement, *p* < 0,001, 95% confidence interval from 0,606 to 0.826) and was considered to be good.

Regarding CI, from the comparison between IOE and final diagnosis a variation was observed in only nine of the negative intraoperative cases. The number of observed agreements was 193 (95,54% of the observations). Diagnostic accuracy, sensitivity and specificity of intraoperative FS are summarized in Table 4 for CI. All the cases positive for the cervical invasion, in intraoperative diagnosis as well as in final diagnoses, presented MI > 50% (Table 4). Even in this case the k Cohen’s analysis was performed (k = 0,668, substantial agreement, *p* < 0,001, 95% confidence interval from 0,468 to 0.868) and was considered to be good. Figure 1(d) shows intra and postoperative CI frequencies.

**Table 4:** Comparison between Intraoperative and Final Report of CI

Intraoperative Examination	Final Diagnosis			
		Pos	Neg	TOTAL
	Pos	10	0	10
	Neg	9	183	192
	TOTAL	19	183	202
*Sensitivity: 52.63%*	*95% CI (28.86 to 75.55%)*			
*Specificity: 100.00%*	*95% CI (98.00 to 100.00%)*			
*Accuracy: 95.54%*	*95% CI (91.71 to 97.94%)*			

Abbreviations: Cervical stromal Involvement (CI)

### Pelvic/Paraortic nodes

We have to precise that numerous changes occurred in the intraoperative surgical and pathological nodal staging guidelines across the time of our study (2012–2018) (e.g. side specific lymphadenectomy with frozen sections, sentinel node biopsy and ultrastaging and/or molecular evaluation by OSNA). However, analyzing our data regarding surgical staging according to FS results, as shown in Table 5, we observed 31/238 positive lymph-nodes (13%) with the higher number of positive nodes detected in the high risk population, according to MAYO criteria (Mariani et al).

**Table 5:** Intraoperative surgical staging procedure acording to IOE reports

IOE FS	PATIENTS	HYS BSO + LYMPHADENECTOMY	HYS BSO	PATIENTS N+	N. NODES EXAMINED	POSITIVE NODES
	–					
HIGH RISK	86	80	6	18	156	27
LOW RISK	116	38	78	4	82	4
*TOTAL*	*202*	*118*	*84*	*22*	*238*	*31*

## Discussion

The focus of this study was to assess the agreement rate between IOE and FD in a series of surgically resected endometrial carcinoma (EC), with a preoperative ambiguous or inconclusive diagnosis by endometrial biopsies and imaging.

We documented that FS in these cases is a useful tool, helping the surgeon to make the right decision in terms of surgical staging. Our data also demonstrated that endometrioid histology has the highest concordance for intraoperative and final pathology interpretation. In detail, we observed that 175 of 180 patients (97.2%) retained their intraoperative classification, demonstrating a good interobserver agreement in the diagnosis of endometrioid histology. The remaining 5 cases were NECs that were intraoperatively misdiagnosed as EA G3, but this type of misdiagnoses did not impact the classification as ‘high-risk’ cancer.

On the other hand, the majority of discordant agreement rates regarded the NEC category. In fact, although the overall agreement was good we found a general discordant rate of 41% for NEC.

We retain that these discrepancies may be explained by the limited dimensions of samples, the lack of a macroscopically appreciable tumoral lesion and the presence of freezing artefacts that could critically affect the discordant rate [9, 10].

Differently from all the authors who demonstrated that in endometrial cancer the intraoperative tumor grade valuation is not a good predictor of final pathology, with an overall agreement range from 30 to 60% in literature [9–15], our data have shown a good grading agreement rate (91,09%) with a global k-index of 0.775. In fact, the intraoperative tumor grade was confirmed in the final diagnosis in 184/202 cases, moreover, no diagnoses were downgraded and 18 cases (8,9%) were upgraded on final pathology report. In particular, we found that the vast majority of shifts occurred from low grade (G1) to intermediate grade (G2) while, none of the G1 tumors shifted its grade in high grade (G3) on final pathology. Only 2 cases intraoperatively diagnosed as G2 shifted in high grade (G3) on permanent sections.

These findings, underline the usual tendency to underestimate rather than overestimate the histological grade of the tumour in an intraoperative setting. One explanation is that pathologists may be prudent of over-diagnosing cancer, in order to avoid extensive surgery and its potential complications for the patient.

In contrast to our study, several papers demonstrated a poor correlation between frozen section and final diagnosis [9–15]. In detail, the lowest agreement occurred in low-grade and minimally invasive lesions. The main reasons for these discrepancies include potential artifacts related to the FS technique and inadequate sampling [16]. In fact, macroscopic determination of the extent of myometrial invasion may be challenging especially in low grade tumors considering also that the invasion line can be heterogeneous with presence of skip metastasis. Moreover, FS has poor sensitivity to detect microscopic neoplastic foci in the cervix, which could be found only in permanent sections [14, 15]. In our study, the higher agreement rates may be explained by the fact that all surgical samples were macroscopically and pathologically evaluated by a specialized gynecologic pathologists. Moreover, when microscopic findings were considered equivocal, additional sections from the fresh specimen were obtained in order to increasy the FS accuracy.

Regarding the intraoperative gross and microscopic examination of the depth of myometrial invasion, it can be considered a relatively fast and accurate method, useful for identifying cancers at high risk for extrauterine metastases [17, 18]. With an 89.1% concordance rate between FS and PS regarding MI assessment, our study confirmed these results. The obtained 10.9% of discordance rate could have an explanation in possible sampling errors, particularly in those tumors macroscopically appearing to be confined to the endometrium. Due to some limits of intraoperative examination (prolonged duration of operation with increased risk of infection and side effects from longer exposure to general anesthesia for the patient and problems in medical and technical resource management), other imaging techniques, in particular the magnetic resonance imaging (MRI), have also been preoperatively employed as alternative tool to evaluate the depth of MI [19, 20]. Although with a good level of accuracy, this technique remains expensive and not always available especially in developing countries and further studies should be aimed to compare the diagnostic accuracy of FS or gross examination with MRI in predicting the degree of myometrial invasion [19–21].

Considering the cervical stromal involvement, it is necessary to remark the numerous possible impediments to obtain an accurate diagnosis of cervical stromal invasion, also occurring on PS: the exact determination of the junction between the lower uterine segment and upper endocervix, the identification of “floaters”, the precise distinction between cervical superficial/glandular involvement and stromal invasion and the distinction between cervical glandular involvement and reactive non-neoplastic lesions of the endocervical glands (embryological remnants and endocervical hyperplasias or metaplasias) [22, 23].

In our study, a variation in CI was observed in only 9 cases and the rate of concordance was 95%. Moreover, considering some recent data suggesting that cervical stromal invasion alone is not independently associated with clinical outcome, [23–25] we believe that in daily practice, a diagnosis of cervical stromal invasion should be evaluated in the entire clinico-pathological context, remembering that CI often co-exists with other factors capable of influencing the prognosis, such as deep myometrial invasion, high tumor grade and the presence of lymphovascular invasion.

Finally, our study has several limitations due to its retrospective nature and the possible data bias. Moreover, our findings demonstrate that 21 patients (10,4%) were intraoperatively misdiagnosed, receiving inappropriate surgical staging (Table 6). In spite of the frequent shifts observed between intraoperative and postoperative histological features, multiple studies have shown that, avoiding lymphadenectomy has no deleterious impact on the overall survival or disease-free survival, in low risk EC patients [26] .

**Table 6:** Misdiagnosed patients

	HIGH RISK	LOW RISK
IOE FS	86	116
FD	107	95

## Conclusions

In conclusion, in the setting of current standards of care, we are aware that actually most centers are performing sentinel lymph node mapping and thus the role of frozen section evaluation appears to be diminishing. Anyway as recently defined by some Authors [27] we have demonstrated that the intraoperative FS could still be considered a useful diagnostic tool, which in few minutes (about 20 min), when performed by a dedicated gynaecological pathologists team, provides accurate information about the risk stratification of EC patients. In particular, IOE is useful in those cases in which the classical histopathological prognostic information are preoperatively ambiguous or inconclusive by imaging and endometrial biopsies (often too small or not well representative of the entire lesions, or rich in necrosis or bloody samples).

## Data Availability

The data belong to the Department of Women and Child Health Database Center and the datasets generated during the current study are not publicly available due to participant identifying factors. They are available from the corresponding author on reasonable request.

## Abbreviations

CI: Cervical Involvement
EA: Endometrioid
EC: Endometrial Carcinoma
FD: Final Diagnosis
FIGO: International Federation of Gynecology and Obstetrics
FS: Frozen section
IOE: Intraoperative Evaluation
MI: Myometrial Invasion
MRI: Magnetic resonance imaging
NEC: Non-Endometrioid Carcinoma
PS: Permanent sections
